# A *Sinorhizobium meliloti*-specific *N*-acyl homoserine lactone quorum-sensing signal increases nodule numbers in *Medicago truncatula* independent of autoregulation

**DOI:** 10.3389/fpls.2014.00551

**Published:** 2014-10-14

**Authors:** Debora F. Veliz-Vallejos, Giel E. van Noorden, Mengqi Yuan, Ulrike Mathesius

**Affiliations:** Department of Plant Science, Research School of Biology, Australian National UniversityCanberra, ACT, Australia

**Keywords:** acyl homoserine lactones, autoregulation of nodulation, ethylene, flavonoids, nodulation, quorum sensing

## Abstract

*N*-acyl homoserine lactones (AHLs) act as quorum sensing signals that regulate cell-density dependent behaviors in many gram-negative bacteria, in particular those important for plant-microbe interactions. AHLs can also be recognized by plants, and this may influence their interactions with bacteria. Here we tested whether the exposure to AHLs affects the nodule-forming symbiosis between legume hosts and rhizobia. We treated roots of the model legume, *Medicago truncatula*, with a range of AHLs either from its specific symbiont, *Sinorhizobium meliloti*, or from the potential pathogens, *Pseudomonas aeruginosa* and *Agrobacterium vitis*. We found increased numbers of nodules formed on root systems treated with the *S. meliloti*-specific AHL, 3-oxo-C_14_-homoserine lactone, at a concentration of 1 μM, while the other AHLs did not result in significant changes to nodule numbers. We did not find any evidence for altered nodule invasion by the rhizobia. Quantification of flavonoids that could act as *nod* gene inducers in *S. meliloti* did not show any correlation with increased nodule numbers. The effects of AHLs were specific for an increase in nodule numbers, but not lateral root numbers or root length. Increased nodule numbers following 3-oxo-C_14_-homoserine lactone treatment were under control of autoregulation of nodulation and were still observed in the autoregulation mutant, *sunn4 (super numeric nodules4)*. However, increases in nodule numbers by 3-oxo-C_14_-homoserine lactone were not found in the ethylene-insensitive *sickle* mutant. A comparison between *M. truncatula* with *M. sativa* (alfalfa) and *Trifolium repens* (white clover) showed that the observed effects of AHLs on nodule numbers were specific to *M. truncatula*, despite *M. sativa* nodulating with the same symbiont. We conclude that plant perception of the *S. meliloti-specific* 3-oxo-C_14_-homoserine lactone influences nodule numbers in *M. truncatula* via an ethylene-dependent, but autoregulation-independent mechanism.

## Introduction

Many species of the legume family interact with nitrogen-fixing bacteria collectively called rhizobia, leading to the formation of root nodules, in which the bacteria are housed. This provides a source of nitrogen to the plant, while the bacteria benefit from a carbon source from the plant host. Rhizobia, like most gram-negative bacteria, synthesize and perceive *N*-acyl-homoserine lactone (AHL) quorum sensing signals (González and Marketon, 2003; Sanchez-Contreras et al., 2007). AHLs contain a homoserine lactone moiety with variable acyl chain length, and different bacterial species produce specific mixtures of AHLs. AHLs mediate a number of cell-to-cell signaling functions in bacteria, and are particularly important for bacteria that interact with plants. Among the traits regulated by AHLs in bacteria, bacterial movement, biofilm formation, production of virulence factors and degradative enzymes have been shown to be important for bacteria-plant interactions (e.g., Parsek and Greenberg, 2000; von Bodman et al., 2003; De Angelis et al., 2008).

In rhizobia, AHLs mediate exopolysaccharide synthesis important for bacterial attachment and invasion, plasmid transfer, swarming behavior, regulation of nitrogen-fixation genes and nodulation efficiency (e.g., Marketon et al., 2002; Wisnieski-Dyé and Downie, 2002; González and Marketon, 2003; Sanchez-Contreras et al., 2007; Cao et al., 2009; Mueller and González, 2011; Gao et al., 2012; Nievas et al., 2012).

While AHLs regulate communication between bacterial cells, there is growing evidence that AHLs are also acting as inter-kingdom signals (Hughes and Sperandio, 2008). Exposure of plants to purified or synthetic AHLs led to the discovery that plants respond specifically to these bacterial signals (Mathesius et al., 2003), and it has been speculated that this perception system may benefit the plant by sensing the presence and activity of nearby bacterial colonies and thus modifying their responses (Bauer and Mathesius, 2004; Teplitski et al., 2011; Hartmann et al., 2014). In support of that hypothesis, a number of studies have demonstrated that AHLs trigger changes in plant development and plant defense (Hartmann et al., 2014). For example, AHLs were shown to alter root architecture in *Arabidopsis thaliana* and mung bean, in part by targeting hormone signaling (Ortíz-Castro et al., 2008; von Rad et al., 2008; Bai et al., 2012; Liu et al., 2012; Zuñiga et al., 2013). In addition, AHLs mediate plant defense responses toward pathogens in tomato and *A. thaliana* (Schuhegger et al., 2006; Schikora et al., 2011; Schenk et al., 2012, 2014; Zarkani et al., 2013). So far, it is not known how plant responses to AHLs alter the interaction of legumes with their rhizobia symbionts.

During the symbiosis of legumes with rhizobia, the plant exudes signal molecules, in most cases flavonoids, into the rhizosphere to attract rhizobia and to induce the expression of nodulation (*nod*) genes in rhizobia (Firmin et al., 1986; Peters et al., 1986; Redmond et al., 1986; Peck et al., 2006). This leads to the synthesis of Nod factors that are necessary for the induction of cell divisions in the host root and the formation of infection threads, leading to the development of an infected nodule (Oldroyd and Downie, 2008). The absence of flavonoids in roots inhibits nodulation (Subramanian et al., 2006; Wasson et al., 2006; Zhang et al., 2009), whereas the addition of external flavonoids acting as *nod* gene inducers has been shown to increase or decrease nodule numbers in legumes, depending on their concentration (Novák et al., 2002). Host-exuded flavonoids have also been shown to increase the production of AHLs in rhizobia, possibly to coordinate the production of AHLs in the vicinity of the host in preparation for successful symbiosis (Pérez-Montaño et al., 2011).

Nodule numbers on the legume root system are under strict control from environmental factors, e.g., nitrogen availability, as well as an internal autoregulation system controlled through receptor-like kinases acting in the shoot (Reid et al., 2011; Mortier et al., 2012). When rhizobia first infect the root system, they induce the formation of regulatory plant peptides of the CLE family, which are transported to the shoot, interact with a receptor-like kinase and thereby generate an inhibitory signal that moves back to the root to limit further nodule initiation (Delves et al., 1986; Okamoto et al., 2013). The receptor-like kinase has been identified in several legumes, including the model legume *Medicago truncatula*, where it was named *SUNN* (*SUPER NUMERIC NODULES*) (Schnabel et al., 2005). The *sunn* mutant and other autoregulation mutants are characterized by the formation of excessive numbers of nodules, typically associated with a smaller shoot and root system (Penmetsa et al., 2003; Schnabel et al., 2005). In addition, nodule numbers are controlled through ethylene signaling, and ethylene-insensitive mutants also show excessive nodule numbers, although this is root- and not shoot-determined. For example, the ethylene insensitive *sickle* mutant of *M. truncatula* hypernodulates, and this is likely due to reduced defense responses during root and nodule infection (Penmetsa and Cook, 1997; Penmetsa et al., 2003, 2008).

In this study, we examined AHL responses in the model legume, *M. truncatula*. In particular, we tested whether AHLs from the rhizobial symbiont of *M. truncatula, Sinorhizobium meliloti*, would exert specific nodulation-related responses in its host. We compared the responses to AHLs known to be synthesized by *S. meliloti* with responses to AHLs known to be synthesized by non-symbiotic bacteria to test if the nodulation responses are specific to recognition of AHLs from the symbiont. We also tested whether the responses are restricted to *M. truncatula* or can be detected in other legumes, as well as whether the responses are specific to nodulation, or whether they extend to alteration of root architecture, as reported from *A. thaliana* (e.g., Ortíz-Castro et al., 2008; Liu et al., 2012).

## Materials and methods

### Plant growth

Seeds of *M. truncatula* wild type (Jemalong A17), its mutants *sunn4* and *skl* as well as *Medicago sativa* cv. Aurora and *Trifolium repens* cv. Haifa were scarified with sand paper, surface-sterilized for 10 min in 6% (v/v) sodium hypochlorite, followed by five washes with sterile water, and soaked for 6 h in a solution containing 200 mg/L amoxycillin and clavulanic acid. This antibiotic treatment reduced bacterial contamination of seedlings to below detection level when seedlings were washed in Lysogeny Broth (LB) to test for contamination. Seeds were stratified for 48 h at 4°C and germinated on Fåhraeus media (FM) agar plates at 21°C for 16 h in darkness (Fåhraeus, 1957). Seedlings were transferred to square petri dishes (245 × 245 × 18 mm) containing FM agar (pH 6.5) with or without AHLs. Ten plants were placed on each plate with three replicates. All plates were incubated in the growth chamber in a complete randomized block design.

Inoculation of *Sinorhizobium meliloti* strain 1021 grown to an OD_600_ of 0.1 in Bergersen's Modified Medium (Rolfe et al., 1980) was performed 3 days after transferring the seedling to the AHL-containing plates. Plants were grown at 25°C at a photosynthetically active radiation (PAR) of 120 μmol m^−2^ s^−1^ for 21 days after inoculation in a controlled temperature room.

For the assay of autoregulation of nodulation the position of the root zone susceptible to infection, i.e., the zone of emerging root hairs just behind the root tip (Bhuvaneswari et al., 1981) was inoculated with *S. meliloti* strain 1021 and this inoculation zone was marked at the back of the petri dish. This zone corresponds to the zone named “0–24 h.” We then marked the susceptible zone again 24 h later at the back of the plate and this zone is corresponds to the 24–48 h post-inoculation “window.” After 21 days, nodule numbers were counted in the marked windows correlating with the positions of the root zone susceptible to nodulation at 0–24 and 24–48 h post-inoculation (Supplementary Figure 1).

To determine nodule biomass, nodules were excised from the root with a scalpel and nodules of 10 plants were pooled as one replicate. In total, three replicates were used. Nodules were immediately weighed to determine their fresh biomass.

The AHLs used (Table 1, Supplementary Figure 2) were purchased from Cayman Chemicals (Ann Arbor, Michigan, USA), dissolved in dimethyl sulfoxide (DMSO) at a concentration of 70 mM and diluted to a final concentration of 1 μM into the FM medium (pH 6.5) following autoclaving and cooling of the medium. Solvent diluted to the same concentration as used for AHLs was used as a negative control.

**Table 1:** **Quorum sensing (QS) signal molecules used in the study and organisms known to synthesize them**.

**QS molecules**	**Organism**	**Reference**
C_4_-HSL	*Pseudomonas aeruginosa*	Pearson et al., 1995
C_6_-HSL	*Sinorhizobium meliloti* AK 631	Teplitski et al., 2003
C_8_-HSL	*S. meliloti* Rm41	Marketon et al., 2002; Teplitski et al., 2003
3-Oxo-C_8_-HSL	*S. meliloti* Rm41	Teplitski et al., 2003
C_10_-HSL	*S. meliloti* AK 631	Teplitski et al., 2003
C_12_-HSL	*S. meliloti* Rm1021	Marketon et al., 2002
3-Oxo-C_12_-HSL	*P. aeruginosa*	Pearson et al., 1994
C_14_-HSL	*S. meliloti* Rm1021	Chen et al., 2003; Teplitski et al., 2003
3-Oxo-C_14_-HSL	*S. meliloti* Rm1021	Teplitski et al., 2003
3-Oxo-C_14:1-7_-cis (L)-HSL	Synthetic AHL analog	Chhabra et al., 2003
C_14:1_-9-cis-(L)-HSL	*Agrobacterium vitis*	Li et al., 2005
C_16_-HSL	*S. meliloti* Rm41	Teplitski et al., 2003
C_16:1_-9 cis-(L)-HSL	*S. meliloti* Rm1021	Marketon et al., 2002
3-Oxo-C_16:1_-11cis-(L)-HSL	*A. vitis*	Hao and Burr, 2006
C_18_-HSL	*S. meliloti* Rm1021	Marketon et al., 2002

### Microscopy

Three day-old *M. truncatula* seedlings were inoculated with the green fluorescent protein (GFP)-expressing *S. meliloti* strain pHC60-GFP (Cheng and Walker, 1998). At 21 days after inoculation, a 0.5 mm long root segment containing nodules was excised from each plant and embedded in 3% agarose. These blocks were sectioned at 100 μm thickness on a vibratome (1000 plus, Vibratome Company, St Louis, MO, USA) and sections arranged in order on a microscope slide. In order to standardize the measurements of the nodule area and infection zone in all the samples, segments with the biggest diameter corresponding to the middle section of each nodule were taken and assessed. The preparations were examined immediately under a Leica Microsystems DM5500 B microscope equipped with epifluorescence detection (Leica, Wezlar, Germany). Two images were taken per sample: One after excitation at 365 nm to visualize the flavonoids visible in the root cortex tissue and the other after excitation at 470 nm to visualize the GFP-fluorescence inside the infected nodule zone. Dual images were overlapped, and the “total nodule area” and the area of the “infection zone” (as indicated in Supplementary Figure 3) were measured and analyzed using Leica LAS 4.4 software (Leica, Wezlar, Germany). The “remaining nodule area” was calculated by subtracting the “infection zone” from the “total nodule area.”

### Quantification of flavonoids in *M. truncatula* roots

The flavonoid content of roots was determined by LC-MS/MS according to Farag et al. (2007) with modifications as specified below. Flavonoids were extracted from wild type *M. truncatula* roots 4 days after exposure to AHLs (or solvent control) and 24 h after inoculation with *S. meliloti* or a mock treatment (bacterial growth medium). For each treatment, a 2 cm long root segment from the root tip upwards (encompassing the root zone susceptible to infection with rhizobia) was excised, weighed on a balance and immediately frozen in liquid nitrogen. For each treatment, 15 root segments were pooled, and five replicates of 15 roots each were independently collected and analyzed. Frozen root tissue was ground in a TissueLyser LT (Qiagen, Hilden, Germany). To each sample, 20 ng of luteolin was added as an internal standard, as luteolin was not detected in *M. truncatula* roots. Flavonoids were extracted with 1 mL of 80% methanol for 14 h at 4°C on a rotator in the dark and centrifuged at 10,000 rpm for 30 min in at 4°C. The supernatants were dried in a Speedvac centrifuge for approximately 60 min. The pellet was resuspended in 45% methanol for analysis.

Flavonoids were separated on an Agilent 6530 Accurate Mass LC-MS Q-TOF (Agilent Technologies, Santa Clara, USA). The samples were run in ESI (electrospray ionization) mode in the Jetstream interface in the negative mode and injected (7 μl) onto an Ascentis® Express 2.7 μm C18 2.1 × 50 mm column (Supelco/Sigma Aldrich, St. Louis, MO, USA). Solvent A consisted of 0.1% aqueous formic acid and solvent B consisted of 90% acetonitrile containing 0.1% formic acid. The elution of the flavonoids was carried out with a linear gradient from 10 to 50% solvent B from 0 to 8 min, 50–70% solvent B from 8 to 12 min (then hold from 12 to 20 min), 70–10% solvent B from 20 to 21 min (then hold from 21 to 30 min) at a flow rate of 200 μl min^−1^. The mode used by the instrument to operate was in extended dynamic mode over a range of m/z 50–1000 using targeted collision induced dissociation (CID; N_2_ collision gas supplied at 18 psi) MS/MS. Naringenin, quercetin and morin were analyzed comparing their respective flavonoid standards from Sigma Chemicals. The mass spectra for biochanin A, medicarpin, daidzein, formononetin, liquitirigenin, isoliquiritigenin, and chrysoeriol in the samples were compared to the MassBank database (Horai et al., 2010). The data analysis was done using Agilent Mass Hunter Workstation Software Qualitative Analysis version B.05.00 (2011).

### Statistical analyses

Different statistical analyses were done depending on the distribution of the data and number of replicates. Instat version 3.06 (Graphpad Software, La Jolla, CA, USA) was used for Kruskall-Wallis tests (with Dunn's post-test), for non-normally distributed data. RStudio version 0.98.501 (R Core Team (2013) was used for Mann-Whitney Wilcoxon tests and Student's *t*-tests for non-normally and normally distributed pairwise comparisons, respectively. Genstat 15th Edition (VSN International, Hemel Hempstead, UK) was used for One-Way ANOVA for normally distributed data. All data were tested for normality and homogeneity of the variance before analysis.

## Results

### Effects of AHLs on nodulation and root architecture of wild type *Medicago truncatula*

To determine whether AHLs modulate the interaction of *M. truncatula* roots with its symbiont, *Sinorhizobium meliloti*, we exposed surface-sterilized, germinated seedlings to 15 different AHLs (Table 1 and Supplementary Figure 2), which are either known to be produced by *S. meliloti* or by other bacteria. We chose some well-studied AHLs synthesized by *Pseudomonas aeruginosa* and *Agrobacterium vitis*, although some of these AHLs may also be produced by other bacteria. Seedlings were placed on sterile FM agar with the addition of 1 μM of each AHLs, or solvent as the control. We chose a concentration of 1 μM because it was within the range of concentrations that were previously shown to elicit plant responses (e.g., Mathesius et al., 2003; Ortíz-Castro et al., 2008; von Rad et al., 2008; Liu et al., 2012; Palmer et al., 2014) and AHL concentrations in the μM to mM range were previously measured in the tomato rhizosphere (Schuhegger et al., 2006).

Seedlings were exposed to AHLs for 3 days before being inoculated with *S. meliloti* strain 1021. Previous experiments showed that major changes in protein accumulation occurred in *M. trucatula* within 24–48 h (Mathesius et al., 2003), and gene expression changes were found within 4 h to 4 days after exposure to AHLs in *A. thaliana* (von Rad et al., 2008), thus we hypothesized that an exposure of 3 days would ensure biological responses to occur in roots prior to inoculation with rhizobia. Nodule numbers were counted and nodule biomass determined 3 weeks post-inoculation. At the same time, we counted the number of emerged lateral roots and determined the tap root length.

**Figure 1:**
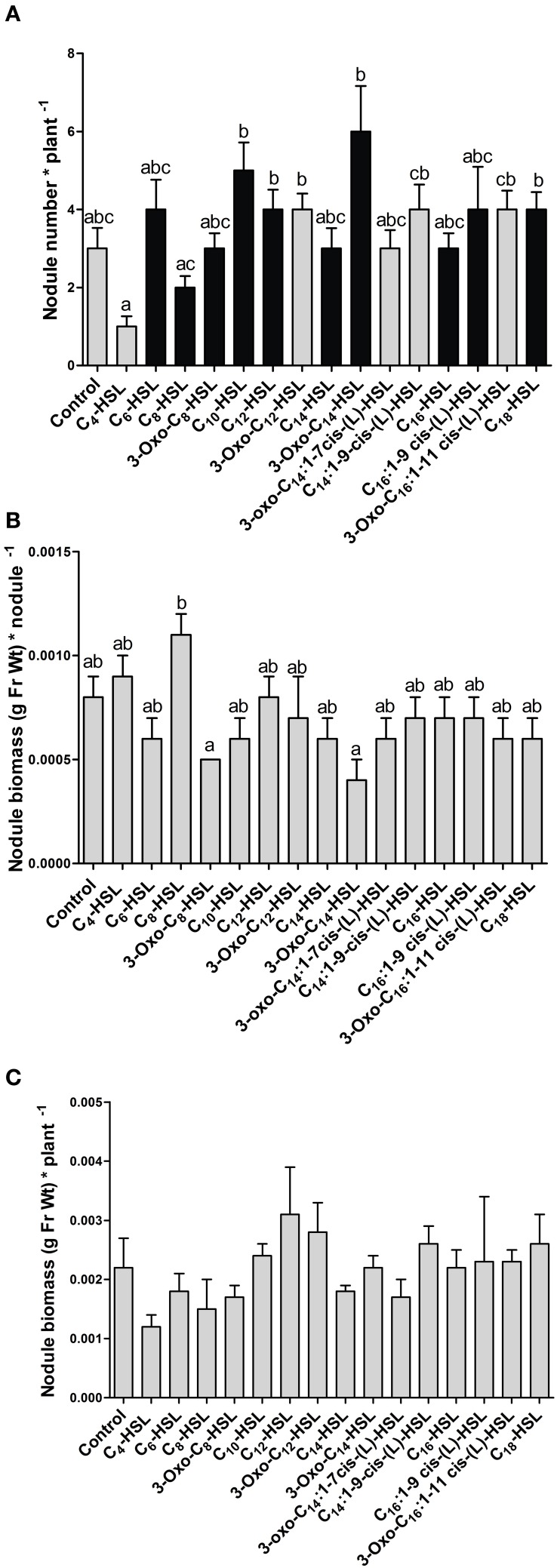
**Effect of 1 μM AHLs on nodulation at 21 days after inoculation in wild type *M. truncatula*. (A)** Nodule numbers per plant; black bars indicate AHLs synthesized by *S. meliloti*, gray bars indicate AHLs synthesized by other bacteria, see Table 1; **(B)** Nodule biomass (in grams of fresh weight) per nodule; **(C)** Nodule biomass (in grams of fresh weight per plant. Data points indicate mean ± *SE*, (*n* = 25–27). **(A)** Kruskall-Wallis test with Dunn's post-test; **(B,C)**: One-Way ANOVA with Tukey post-test at *p* < 0.05. Treatments in **(A,B)** that do not share a common letter are significantly different at *p* < 0.05. No significant differences were found in **(C)**.

AHL exposure led to significant differences in the numbers of nodules between treatments (Figure 1A). However, there was no clear trend toward increased nodule numbers with *S. meliloti*-specific AHLs compared to AHLs from other bacteria. The *S. meliloti* AHL, 3-oxo-C_14_-HSL (homoserine lactone) caused the highest increase in nodule numbers, with almost double the numbers of nodules per plant compared to the control. While this difference was not statistically significantly different from the control, it was repeated in other independent experiments (compare **Figure 4**). We also determined nodule weight per plant and per nodule, which showed that the 3-oxo-C_14_-HSL treatment resulted in the lowest nodule biomass per nodule (Figure 1B), suggesting that nodule numbers in this treatment were increased at the expense of nodule biomass. Differences in the nodule biomass per plant (Figure 1C) showed the same trend as nodule numbers per plant, although none of the treatment differences were statistically significant (*p* > 0.05).

**Figure 2:**
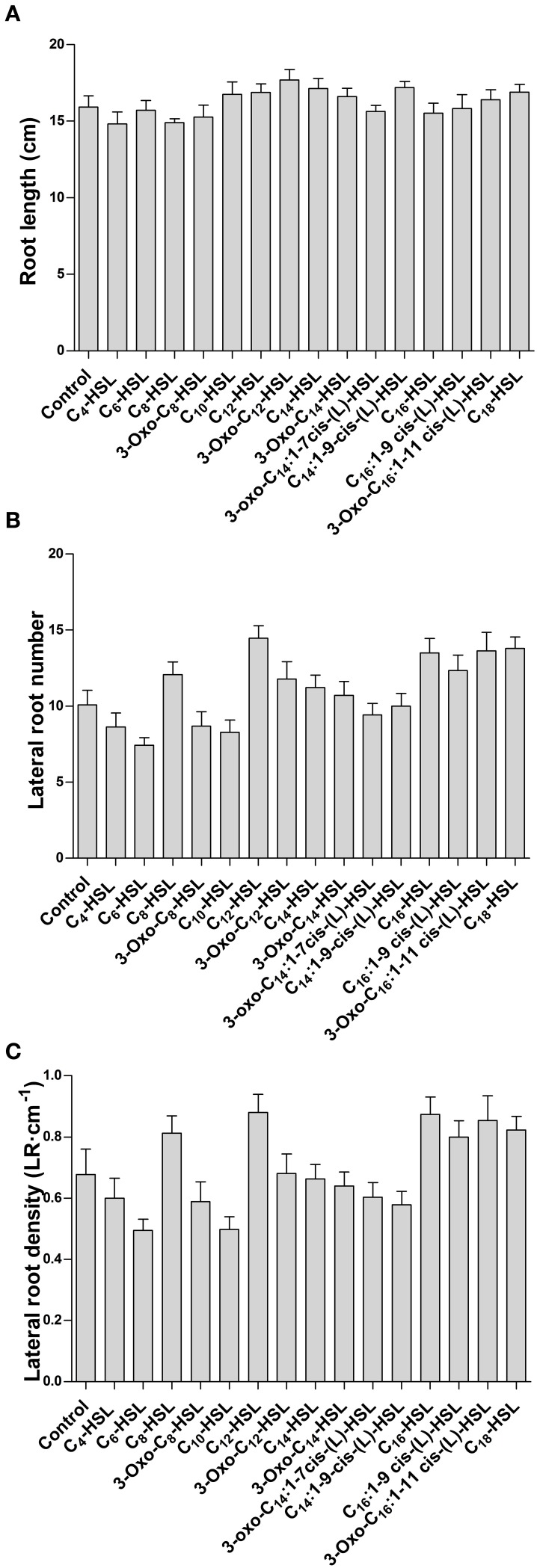
**Effect of AHLs on *M. truncatula* root architecture**. Treatments correspond to 21 days after inoculation in wild type *M. truncatula* treated with 1 μM AHL. **(A)** Root length; **(B)** Lateral root number per plant; and **(C)** Lateral root density. No significant differences at *p* < 0.05 (One-Way ANOVA with Tukey post-test). Data points indicate mean ± *SE*, (*n* = 25–30).

To test whether the effects on nodule numbers were linked to other aspects of root development, we determined lateral root numbers and root length, two phenotypes that are modulated by AHLs in *A. thaliana* at concentrations from around 1–100 μM (e.g., Ortíz-Castro et al., 2008; von Rad et al., 2008; Liu et al., 2012). However, in *M. truncatula* we found no significant changes in root length, lateral root number or lateral root density between 1 μM treatments of the different AHLs (Figures 2A–C).

**Figure 3:**
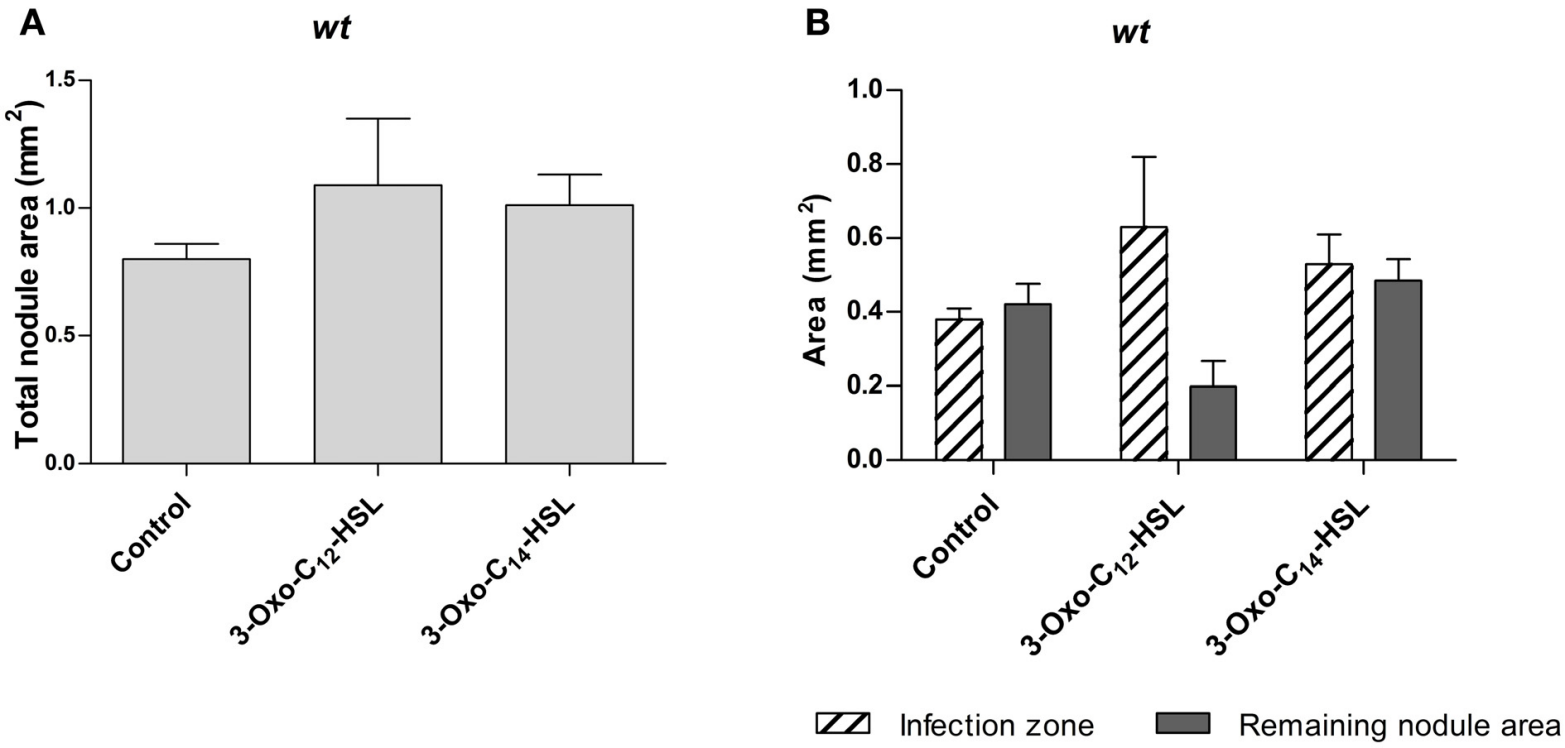
**Nodule area of *M. truncatula* treated with 1 μM AHLs**. Treatments correspond to 21 days after inoculation in wild type *M. truncatula* treated with 3-oxo-C_12_-HSL and 3-oxo-C_14_-HSL. **(A)** Total nodule area; **(B)** infection zone and remaining nodule area. No significant differences at *p* < 0.05 (One-Way ANOVA with Tukey post-test). Data points indicate mean ± *SE*, (*n* = 5–8).

To find out if AHLs alter nodule occupancy by rhizobia, we repeated the experiment with an *S. meliloti* strain expressing a constitutive GFP marker. We tested two AHLs, 3-oxo-C_14_-HSL from *S. meliloti*, which led to increased nodule numbers, and 3-oxo-C_12_-HSL from *P. aeruginosa*, which did not alter nodule numbers. We sectioned 3 week-old nodules and measured the uninfected and infected nodule area in a section through the center of each nodule (Supplementary Figure 3). We found no statistically significant differences in total nodule area, or infected nodule area between treatments (Figure 3).

### Effects of AHLs on nodulation in autoregulation and hypernodulation mutants of *M. truncatula*

**Figure 4:**
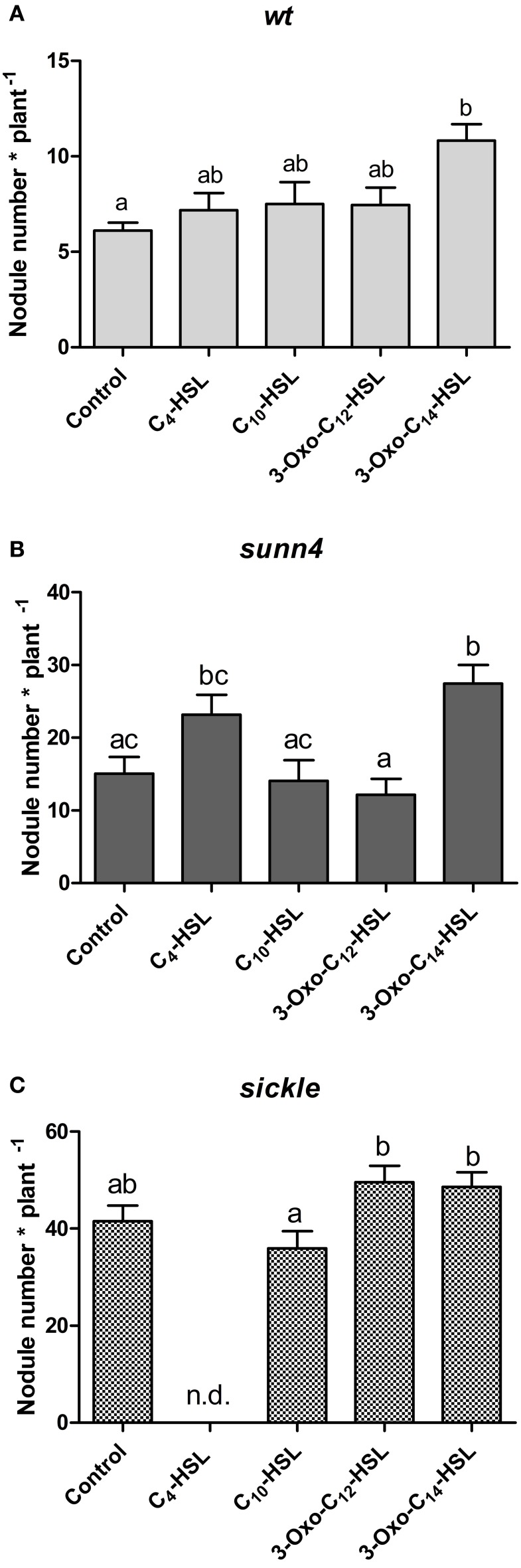
**Nodule numbers of supernodulating mutants at 21 days after inoculation. (A)** wild type (A17); **(B)** *sunn4* mutant; **(C)** *sickle* mutant treated with 1 μM of the indicated AHLs. Data points indicate mean ± *SE* (*n* = 25–30). Treatments that do not share a common letter are significantly different at *p* < 0.05 (**A,B**: Kruskall-Wallis test with Dunn's post-test; **C**: One-Way ANOVA with Tukey post-test). n.d., no determined.

To confirm the effects of AHLs on nodule numbers, we continued our assays with only four AHLs that showed the most prominent changes in nodule numbers in the initial screening experiment (*cf*. Figure 1A). We selected C_10_-HSL and 3-oxo-C_14_-HSL from *S. meliloti* and C_4_-HSL and 3-oxo-C_12_-HSL from *P. aeruginosa*. Of these, only 3-oxo-C_14_-HSL, which showed the highest nodule numbers previously, caused a statistically significant increase in nodule numbers in wild type seedlings (Figure 4A).

**Figure 5:**
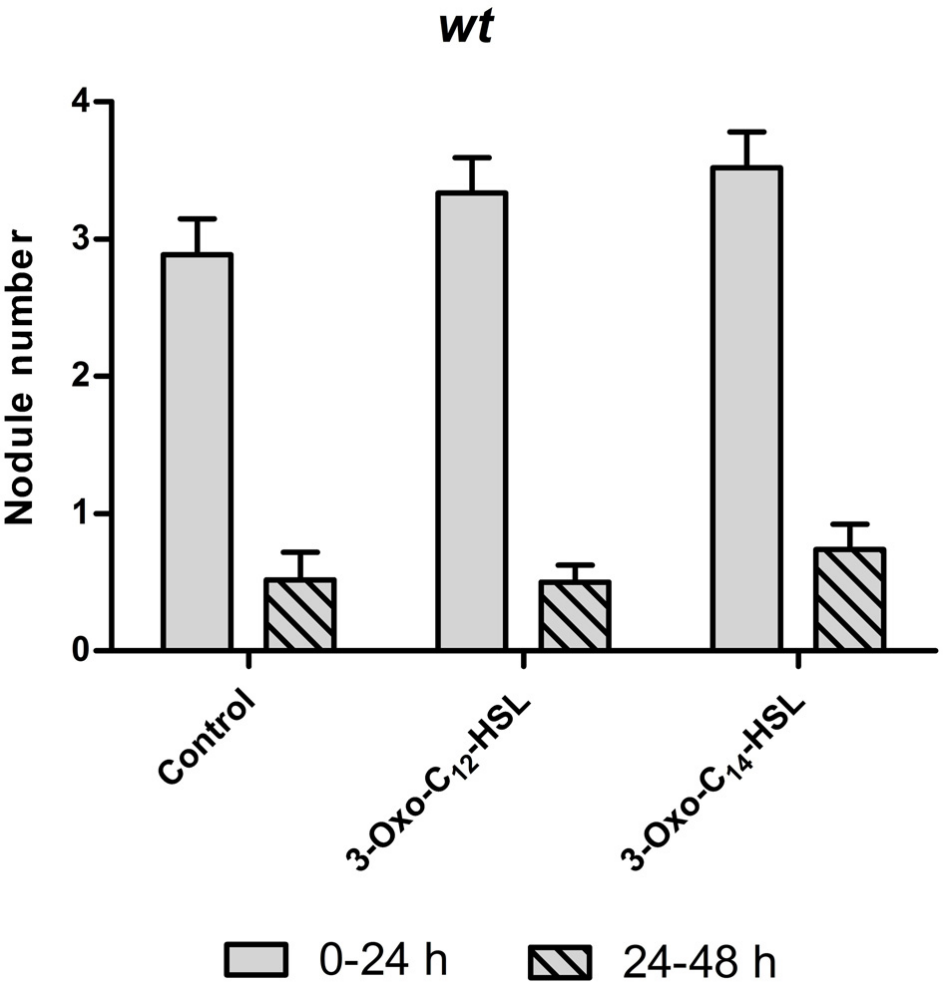
**Autoregulation of nodulation (AON) in wild type *Medicago truncatula* seedlings**. Number of nodules formed after inoculation at two time points (0–24 h) and (24–48 h), for experimental setup see Supplementary Figure 1. No significant differences at *p* < 0.05 (Kruskall-Wallis test with Dunn's post-test). Data points indicate mean ± *SE*, (*n* = 26–30).

One possibility for increased nodule numbers observed in the treatment with 3-oxo-C_14_-HSL, would be that this AHL inhibits the reduction of nodule numbers by the systemic autoregulation of nodulation (AON) mechanism, or by circumventing nodule inhibition by ethylene. To test this we first counted nodule numbers on the root of wild type seedlings in the root segment corresponding to the nodulation zones at 0–24 h and 24–48 h after inoculation with *S. meliloti* (Supplementary Figure 1). Because of AON, nodules are initiated on roots of *M. truncatula* during first 24 h post-inoculation, where after nodule numbers are reduced by systemic AON (van Noorden et al., 2006). We found that treatment of roots with either 3-oxo-C_12_-HSL or with 3-oxo-C_14_-HSL led to the expected reduction of nodule numbers in the 24–48 h window, suggesting that the AHL treatment does not prevent AON (Figure 5).

To further validate this result, we tested the effect of the selected subset of AHLs on nodule numbers in the systemic autoregulation mutant *sunn4*. We found a significant increase in nodule numbers following treatment with 3-oxo-C_14_-HSL in *sunn4* mutant, similar to the wild type (Figures 4A,B), suggesting that this AHL increased nodule numbers independent of AON.

A further possibility to explain increased nodule numbers by AHLs is that AHL treatment alters ethylene signaling. To test this we carried out nodulation experiments in the ethylene-insensitive *skl* mutant. We found that in this mutant, the significant increase in nodule numbers following 3-oxo-C_14_-HSL treatment was lost (Figure 4C). However, nodule numbers in both 3-oxo-C_14_-HSL- and control-treated *skl* mutants were a lot higher than in wild type or *sunn4* mutants roots and may have reached a maximum for the root system. This suggests that ethylene signaling might be required for the increase in nodule numbers following 3-oxo-C_14_-HSL exposure, but further experiments would need to confirm this hypothesis.

### Effects of AHLs on flavonoid production by *M. truncatula* wild type

We further tested whether the alteration in nodule number following AHL treatment could be due to a different root flavonoid profile to increase *nod* gene inducing or *nod* gene inhibiting flavonoids. We quantified the amounts of flavonoids extracted from roots of *M. truncatula* exposed to a subset of the previously tested AHLs (with C_4_-HSL, C_10_-HSL, 3-oxo-C_12_-HSL, and 3-oxo-C_14_-HSL) using LC-MS/MS. Of these, only 3-oxo-C_14_-HSL had led to significant increased in nodule numbers (*cf*. Figure 4A).

**Figure 6:**
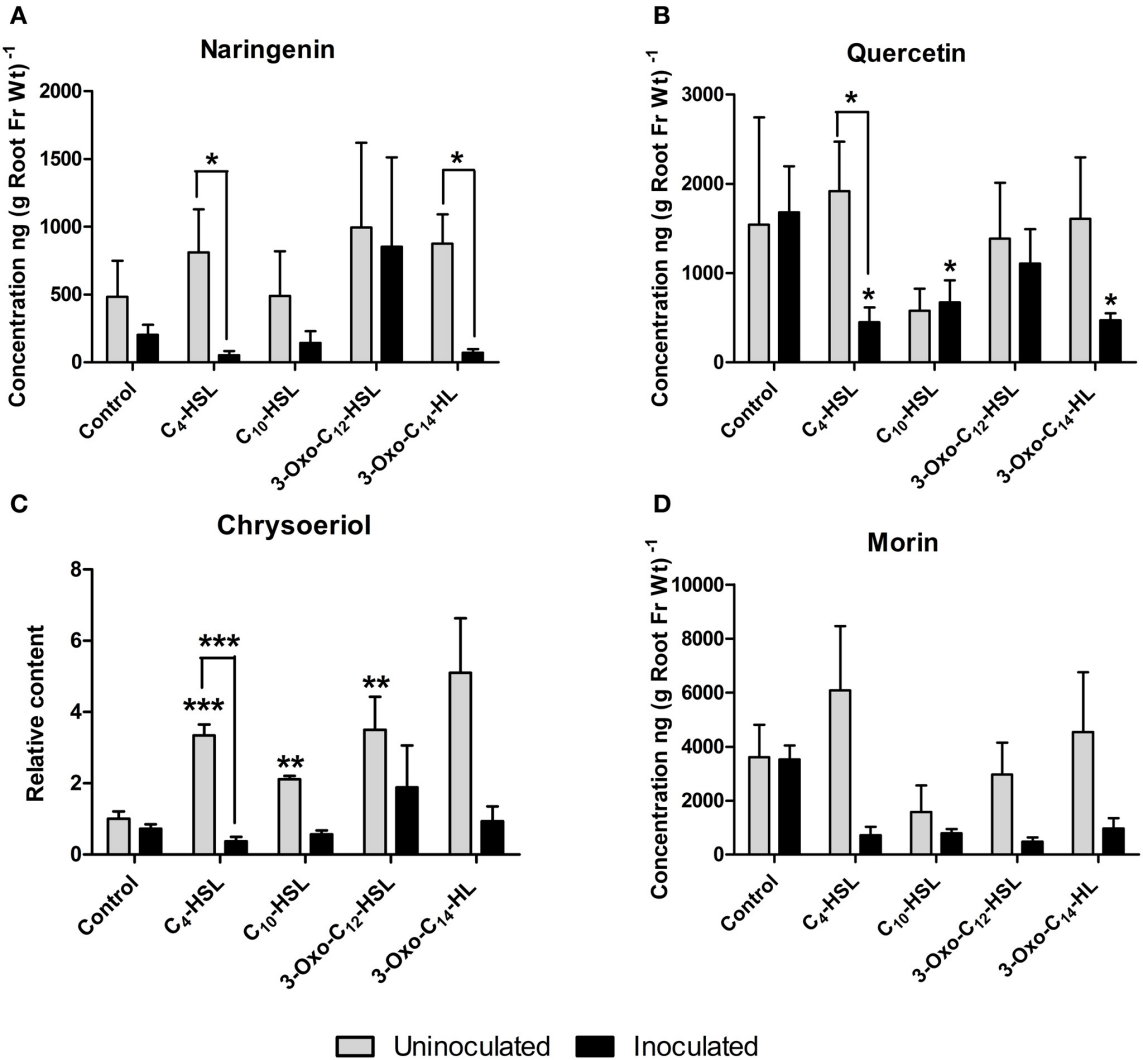
**Effect of AHLs on flavanone, flavonol and flavone content in roots of *M. truncatula* 4 day-old seedlings treated with 1 μM AHLs. (A)** Naringenin (flavanone) **(B)** Quercetin (flavonol), **(C)** Chrysoeriole (flavone), and **(D)** Morin (flavonol). Significant differences between the treatments and the respective control are indicated with asterisks. ^***^Indicates significant differences at *p* < 0.001, ^**^p < 0.01, ^*^p < 0.05 (Student's *t*-test and Mann-Whitney-Wilcoxon test). Data points indicate mean ± *SE* (*n* = 5), i.e., five batches of roots with approximately 20 root segments per batch. g Root Fr Wt^−1^ indicates gram per root fresh weight of the extracted root segments.

**Figure 7:**
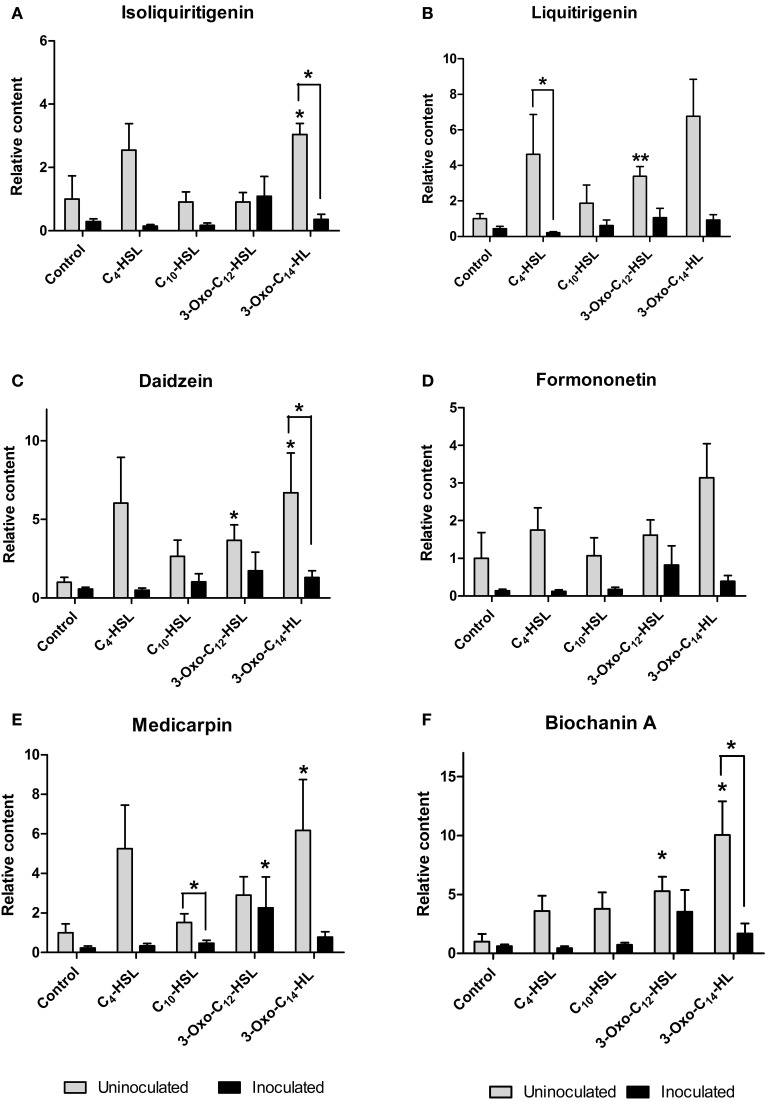
**Effect of AHLs on isoflavonoid content of 4 day-old *M. truncatula*, seedlings treated with 1 μM AHLs. (A)** Isoliquiritigenin **(B)** Liquiritigenin **(C)** Daidzein **(D)** Formononetin **(E)** Medicarpin **(F)** Biochanin A. Significant differences between the treatments and the respective control are indicated with asterisks. ^**^Indicates significant differences at *p* < 0.01 and ^*^indicates significant differences at *p* < 0.05 level (Student's *t*-test and Mann-Whitney-Wilcoxon test). Data points indicate mean ± *SE* (*n* = 5), i.e., five batches of roots with approximately 20 root segments per batch.

Roots were grown on AHL-containing nutrient agar for 24 h before inoculation or mock-inoculation with *S. meliloti* strain 1021 and harvested for analysis 24 h after inoculation. At this time point, flavonoid and auxin responses associated with nodule initiation have previously been detected in *M. truncatula* (Mathesius et al., 2003; Wasson et al., 2006; van Noorden et al., 2007). We found that the flavonoids changed in relative abundance following AHL application, and that there were strong differences between *S. meliloti*-inoculated and mock-inoculated roots (Figures 6, 7). We detected the *nod* gene inducers chrysoeriol (Figure 6C) and isoliquiritigenin (Figure 7A) and the *nod* gene repressor medicarpin (Figure 7E) (Hartwig et al., 1990; Zuanazzi et al., 1998). The concentrations of chrysoeriol and isoliquiritigenin did not increase in inoculated and AHL-treated roots, even though their concentrations did increase after AHL treatment alone. The concentration of medicarpin was significantly increased in 3-oxo-C_12_-treated roots compared to solvent control treated roots, but this did not correlate with a change in nodule numbers in the 3-oxo-C_12_-HSL treatment. The concentrations of the flavonol quercetin, which could act as an auxin transport inhibitor during nodulation (Zhang et al., 2009), was significantly reduced in inoculated roots treated with C_4_-HSL, C_10_-HSL, and 3-oxo-C_14_-HSL. However, only one of these treatments, 3-oxo-C_14_-HSL, increased nodule numbers. Overall, these results do not point to an increase in *nod* gene inducing flavonoids or a decrease in *nod* gene repressing flavonoids in *S. meliloti*-infected roots treated with AHLs that increased nodule numbers (3-oxo-C_14_-HSL) compared to those that did not. Similarly, no increase in flavonols that could act as auxin transport inhibitors during nodulation (Zhang et al., 2009) was found in treatments that increased nodule numbers.

Interestingly, AHL treatment in the absence of rhizobia led to the induction of several of the isoflavonoids and their precursors (liquiritigenin, daidzein, biochanin A and medicarpin; Figure 7) and the concentration of the flavone chrysoeriol (Figure 6C), while inoculation with *S. meliloti* generally attenuated the increases in flavonoid concentrations (Figure 7).

### Effects of AHLs on nodulation and root architecture of different legumes

**Figure 8:**
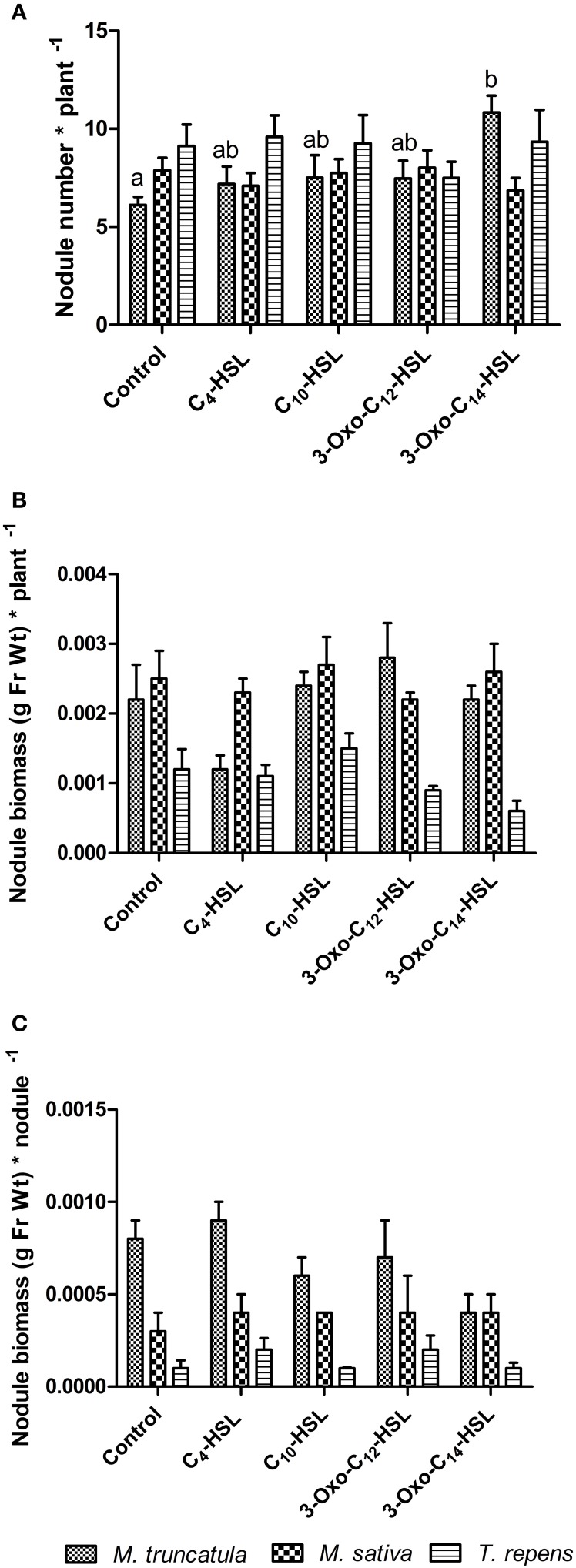
**Effect of AHLs on nodulation in legume species at 21 days after inoculation: *M. truncatula, M. sativa*, and *T. repens* treated with 1 μM AHLs**. **(A)** Nodule number per plant; **(B)** Nodule biomass (in grams of fresh weight) per plant; **(C)** Nodule biomass (in grams of fresh weight) per nodule. Data points indicate mean ± *SE* (*n* = 25–30). In **(A)**, treatments of *M. truncatula* plants that do not share a common letter are significantly different at *p* < 0.05 (One-Way ANOVA with Tukey post-test). Differences in **(B,C)** were not significant.

**Figure 9:**
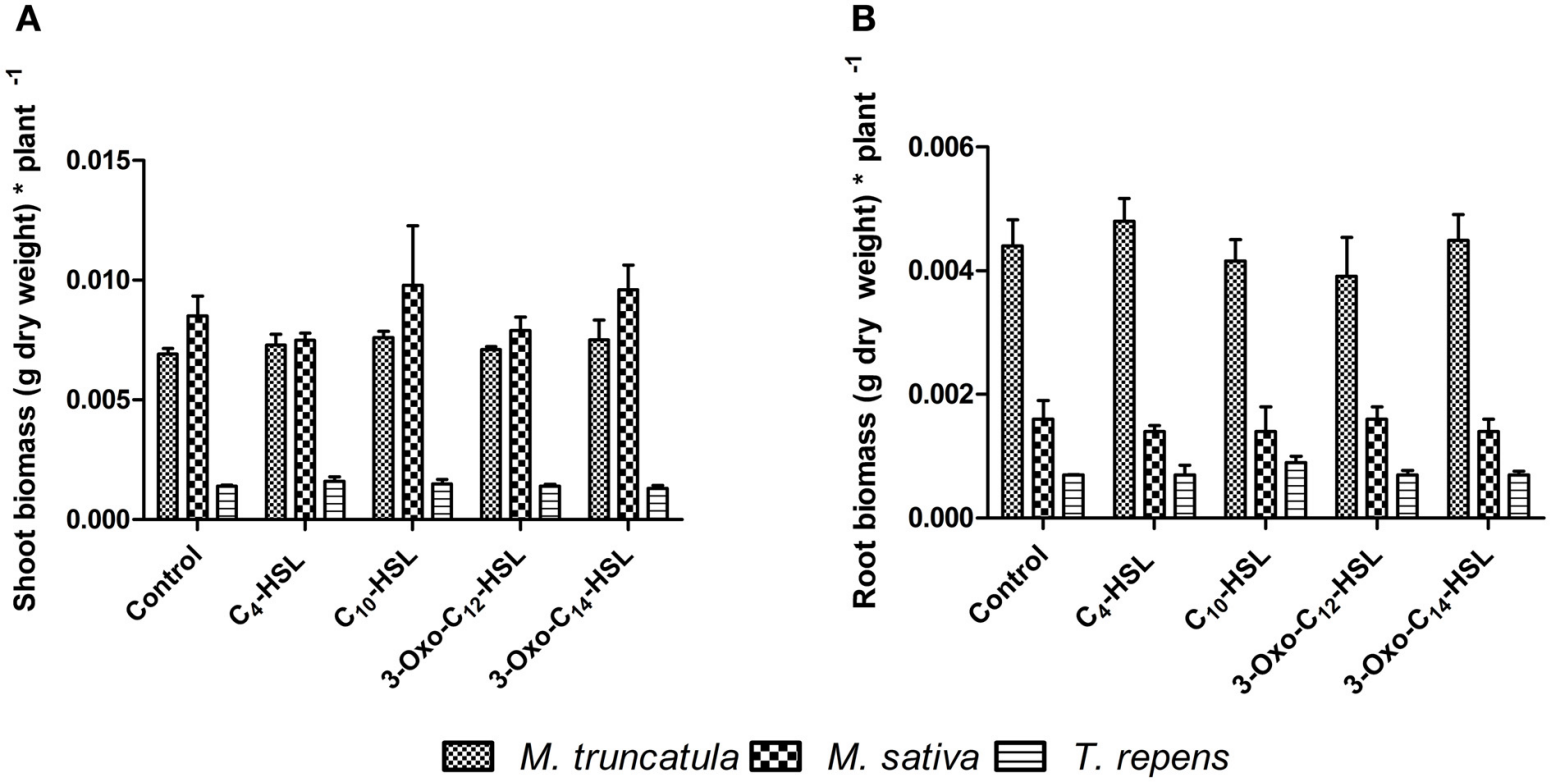
**Effect of AHLs on the shoot and root biomass of different legume species at 21 days after inoculation: *M. truncatula, M. sativa*, and *T. repens* treated with 1 μM AHLs. (A)** Shoot dry biomass (g) **(B)** Root dry biomass (g). Data points indicate mean ± *SE* (*n* = 25–30). No significant differences at *p* < 0.05 level (One-Way ANOVA with Tukey post-test).

**Figure 10:**
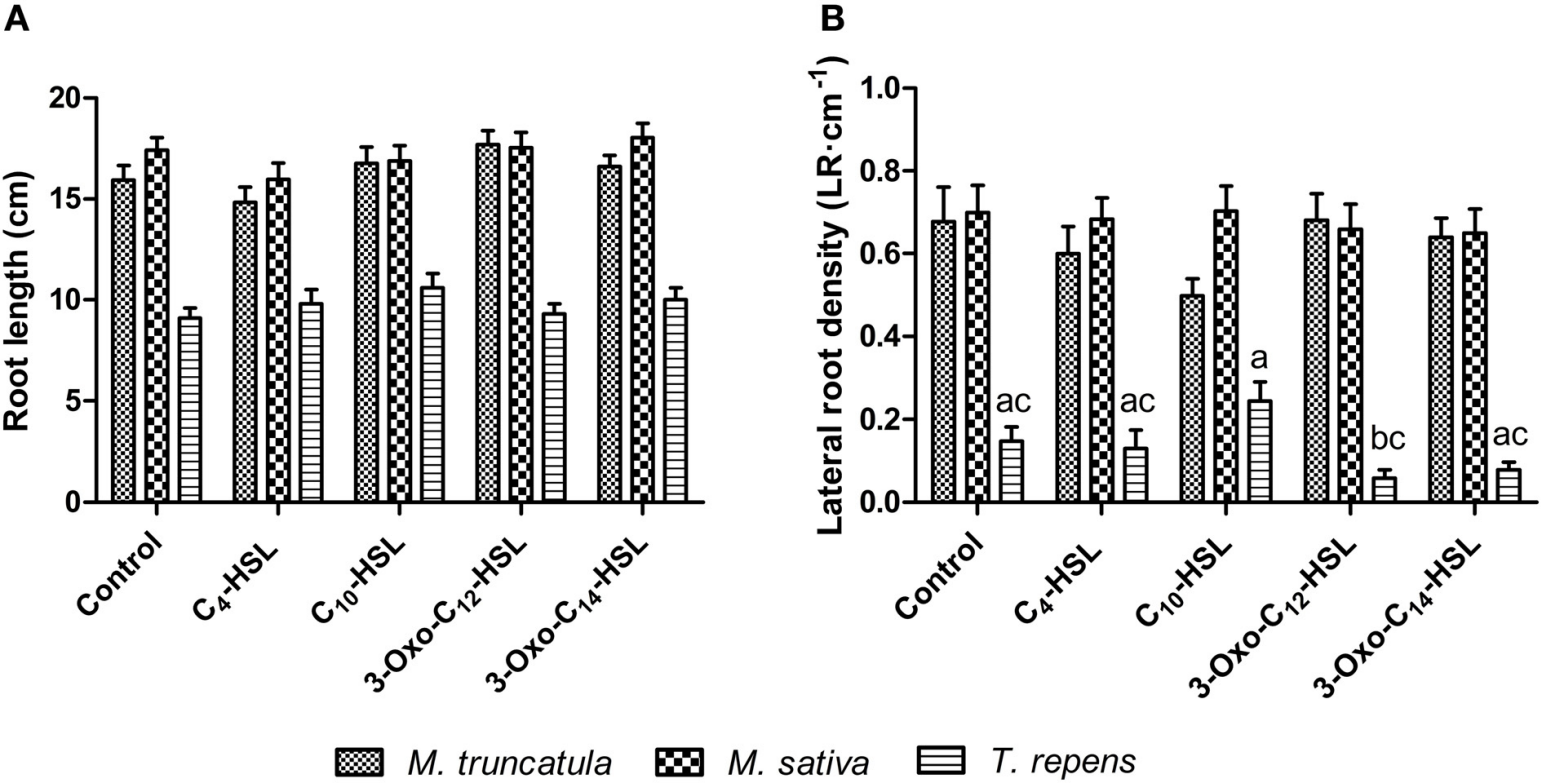
**Effect of AHLs on root architecture of different legume species at 21 days after inoculation: *M. truncatula, M. sativa*, and *T. repens* treated with 1 μM AHLs**. **(A)** Root length, **(B)** Lateral root density. Data points indicate mean ± *SE* (*n* = 25–30). In **(B)**, treatments of *T. repens* plants that do not share a common letter are significantly different at *p* < 0.05 (One-Way ANOVA with Tukey post-test).

To test whether the observed responses on nodule numbers and root architecture in *M. truncatula* were conserved in other legumes, we conducted an experiment in which we compared *M. truncatula, M. sativa* (alfalfa), which also nodulates with *S. meliloti*, and *T. repens* (white clover), which nodulates with *Rhizobium leguminosarum*, bv. *trifolii*. Interestingly, only *M. truncatula* showed significant differences in nodule numbers between AHL treatments (1 μM), with no significant effects in the other two legumes (Figure 8A). There were no significant differences in nodule biomass per plant (Figure 8B), nodule biomass per nodule (Figure 8C), or shoot and root dry biomass (Figure 9) in any of the three legumes. Root length was also unaffected by AHL treatment (Figure 10A), whereas lateral root density was significantly altered by AHLs in *T. repens*, but not *M. truncatula* or *M. sativa* (Figure 10B).

## Discussion

This study aimed at finding out whether exposure of legumes to AHLs from their symbionts, as opposed to those from non-symbionts, alters nodulation. This question arose from findings that plants exposed to AHLs specifically alter gene and protein expression (e.g., Mathesius et al., 2003; von Rad et al., 2008), suggesting that plants interpret signals from surrounding bacteria that could alter the outcome of plant-microbe interactions (Hartmann et al., 2014). For example, exposure of plants to AHLs has been shown to alter the outcome of plant-pathogen interactions (e.g., Schuhegger et al., 2006; Schikora et al., 2011; Schenk et al., 2014).

We found that most AHLs tested, whether synthesized by *S. meliloti* or other bacteria, had no significant effect on nodule numbers in *M. truncatula* at the tested concentration (1 μM). However, one of the AHLs specifically synthesized by its symbiont *S. meliloti*, 3-oxo-C_14_-HSL, repeatedly increased nodule numbers on *M. truncatula* roots. This increase was accompanied by a reduction in nodule biomass, but nodules appeared normal in their infection with rhizobia. The effect was specific to an increase in nodule numbers, while lateral root numbers and root length were not altered. While lateral roots and nodules show similarities in their initiation from root precursor cells, they also show differences in hormonal regulation and cell types involved in their organogenesis (Hirsch and LaRue, 1997; Mathesius, 2008). At the time point measured (3 weeks post-inoculation), root and shoot biomass showed no changes, although it is possible that nodule number changes only result in changes to biomass after a longer time interval.

Interestingly, a comparison of nodulation phenotypes in three legume species showed that at the concentration of 1 μM used in these experiments, only *M. truncatula* showed significant changes in nodule numbers in response to AHLs, despite *M. sativa* nodulating with the same symbiont. It is possible that *M. sativa* responds more or less sensitively to AHLs, and future experiments could test a range of AHL concentrations in legumes to determine whether species show differences in the sensitivity to AHLs. The fact that *T. repens* responded with changes in lateral root numbers to 3-oxo-C_12_-HSL, while the other two legumes did not, suggests that different root phenotypes could respond to different thresholds and/or structures of AHLs. *A. thaliana* strongly responds to certain AHLs with changes in root growth and lateral root numbers (Ortíz-Castro et al., 2008; Bai et al., 2012; Liu et al., 2012; Palmer et al., 2014). Experiments in *M. truncatula* in our lab have shown that the AHL C_10_-HSL, which strongly reduces root growth in *A. thaliana*, does not inhibit root growth in *M. truncatula* at concentrations of 1 or 10 μM (D. Veliz-Vallejos, unpublished results), suggesting that these responses are species-specific. Certain AHLs did affect root elongation in *M. truncatula* in a study by Palmer et al. (2014), and this, as well as other AHL responses, was highly concentration dependent.

In an attempt to find out whether the increase in nodule numbers resulting from plant exposure to 3-oxo-C_14_-HSL was due to altered flavonoid induction in the host we quantified flavonoids in uninoculated and inoculated roots. Several of the isoflavonoids significantly increased after root exposure to AHLs, but not when roots were inoculated with rhizobia at the same time. This supports earlier data on the induction of isoflavone reductase by AHLs in *M. truncatula* using proteomics (Mathesius et al., 2003). AHL-treated and *S. meliloti* inoculated roots did not show any increase or decrease in flavonoids in the roots that correlated with increased nodule numbers in 3-oxo-C_14_-HSL-treated roots. Therefore, currently there is no evidence that altered flavonoid profiles in the host roots could explain the alteration in nodule numbers in response to 3-oxo-C_14_-HSL.

We further used nodulation mutants of *M. truncatula* that are either defective in autoregulation of nodulation (AON), i.e., the autoregulation system reducing nodule numbers through systemic signaling (Schnabel et al., 2005), or in ethylene signaling involved in regulation of nodule numbers through effects on defense responses (Penmetsa and Cook, 1997; Penmetsa et al., 2003, 2008). The AHL 3-oxo-C_14_-HSL still significantly increased nodule numbers in the AON mutant, *sunn4*. This agreed with a temporary reduction of nodule numbers after 24 h post-inoculation onward in 3-oxo-C_14_-HSL- as well as solvent control treated wild type roots, the time window when the autoregulation signal is expected to travel from the shoot to the root to inhibit nodulation (van Noorden et al., 2006). This suggests that the increased numbers of nodules following 3-oxo-C_14_-HLS-treated roots are not a result of an inhibition of AON by this AHL. However, 3-oxo-C_14_-HSL was not able to significantly increase nodule numbers in the ethylene-insensitive *skl* mutant, suggesting that the increase in nodule numbers, at least partly, involves ethylene signaling through EIN2. Ethylene is a negative regulator of nodulation, so that it is most likely that 3-oxo-C_14_-HSL down-regulates ethylene signaling to increase nodule numbers. However, because of the already high number of nodules in the *skl* mutant, future experiments could test whether application of various concentrations of ethylene inhibitors would result in a similar negation of AHL exposure on nodulation. In *A. thaliana* the inhibition of root length by 3-oxo-C_12_-HSL and the breakdown product L-homoserine could be rescued by application of the ethylene synthesis inhibitor, AVG (aminoethoxyvinyl glycine), and similarly in the *M. truncatula skl* mutant, which was the same mutant as used in this study, suggesting that ethylene mediates these root growth responses (Palmer et al., 2014).

Interestingly, 3-oxo-C_14_-HSL from *S. meliloti* was shown to specifically enhance the resistance of *A. thaliana* toward the pathogens *Golovinomyces orontii* and *Pseudomonas syringae*, and resistance of *Hordeum vulgare* (barley) to *Blumera graminis* (Schikora et al., 2011; Schenk et al., 2012; Zarkani et al., 2013). Collectively these studies indicate a specific role for 3-oxo-C_14_-HSL in modulation of host defense responses that could alter the outcome of both pathogenic and symbiotic plant-microbe interactions. Further studies are necessary to investigate the mechanism of how this is achieved. We currently do not know whether the added AHLs directly affect the plant or whether indirect effects via altered perception of AHLs in the symbiont play a role. In addition, it is likely that the AHLs that show effects on plants are processed before or after they are first perceived, e.g., by enzymatic degradation by the plant host (Palmer et al., 2014). One of the breakdown products of AHLs, L-homoserine, has been shown to affect root length in *A. thaliana* (Palmer et al., 2014), and it would be interesting to test its effect on nodulation in future studies. It will also be necessary in the future to determine the detailed perception pathway of AHLs in *M. truncatula*, as well as their molecular mechanism of action responsible for changes in nodulation.

## Author contributions

Conception of the work: Debora F. Veliz-Vallejos, and Ulrike Mathesius; Acquisition and analysis of data: Debora F. Veliz-Vallejos, Mengqi Yuan, and Giel E. van Noorden; Interpretation of data Debora F. Veliz-Vallejos, Giel E. van Noorden, Mengqi Yuan, and Ulrike Mathesius; manuscript preparation and revision: Debora F. Veliz-Vallejos, Giel E. van Noorden, Mengqi Yuan, and Ulrike Mathesius. All authors approve the submitted version and agree to be accountable for all aspects of the work.

### Conflict of interest statement

The authors declare that the research was conducted in the absence of any commercial or financial relationships that could be construed as a potential conflict of interest.

## Abbreviations

AHL: acyl homoserine lactone
ANOVA: analysis of variance
AON: autoregulation of nodulation
FM: Fåhraeus media
HSL: homoserine lactone
LC-MS/MS: liquid chromatography—tandem mass spectrometry
QS: quorum sensing.
