# Supplementary material for: A Sinorhizobium meliloti-specific N-acyl homoserine lactone quorum-sensing signal increases nodule numbers in Medicago truncatula independent of autoregulation
Source: Front Plant Sci. 2014 Oct 14;5:551. doi: 10.3389/fpls.2014.00551 (PMC4196514; doi:10.3389/fpls.2014.00551)
Supplement: Supplementary file 1 [file Image1.PDF]

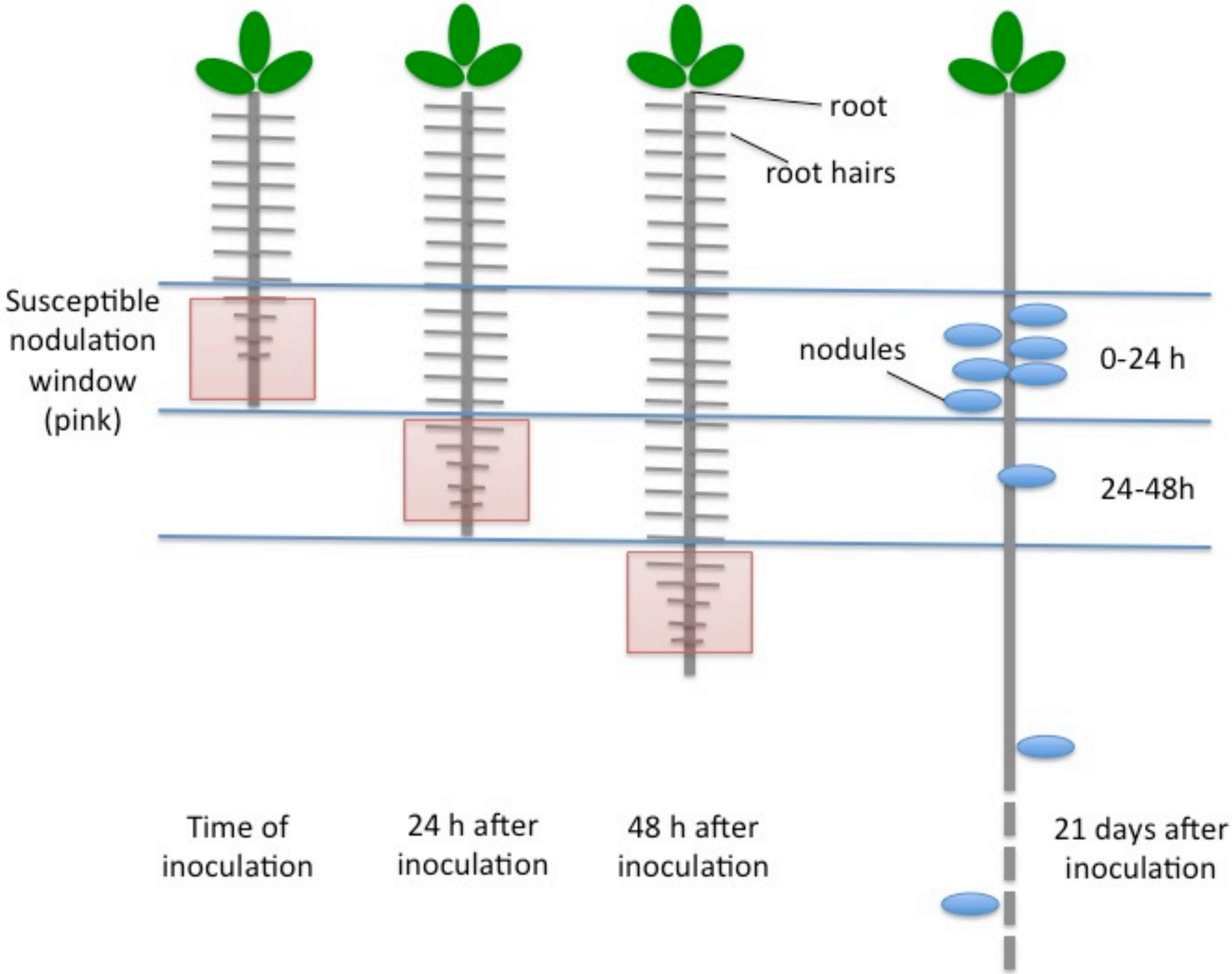


**Supplementary Figure 1**: Schematic diagram of the determination of nodule numbers at different time points after inoculation.

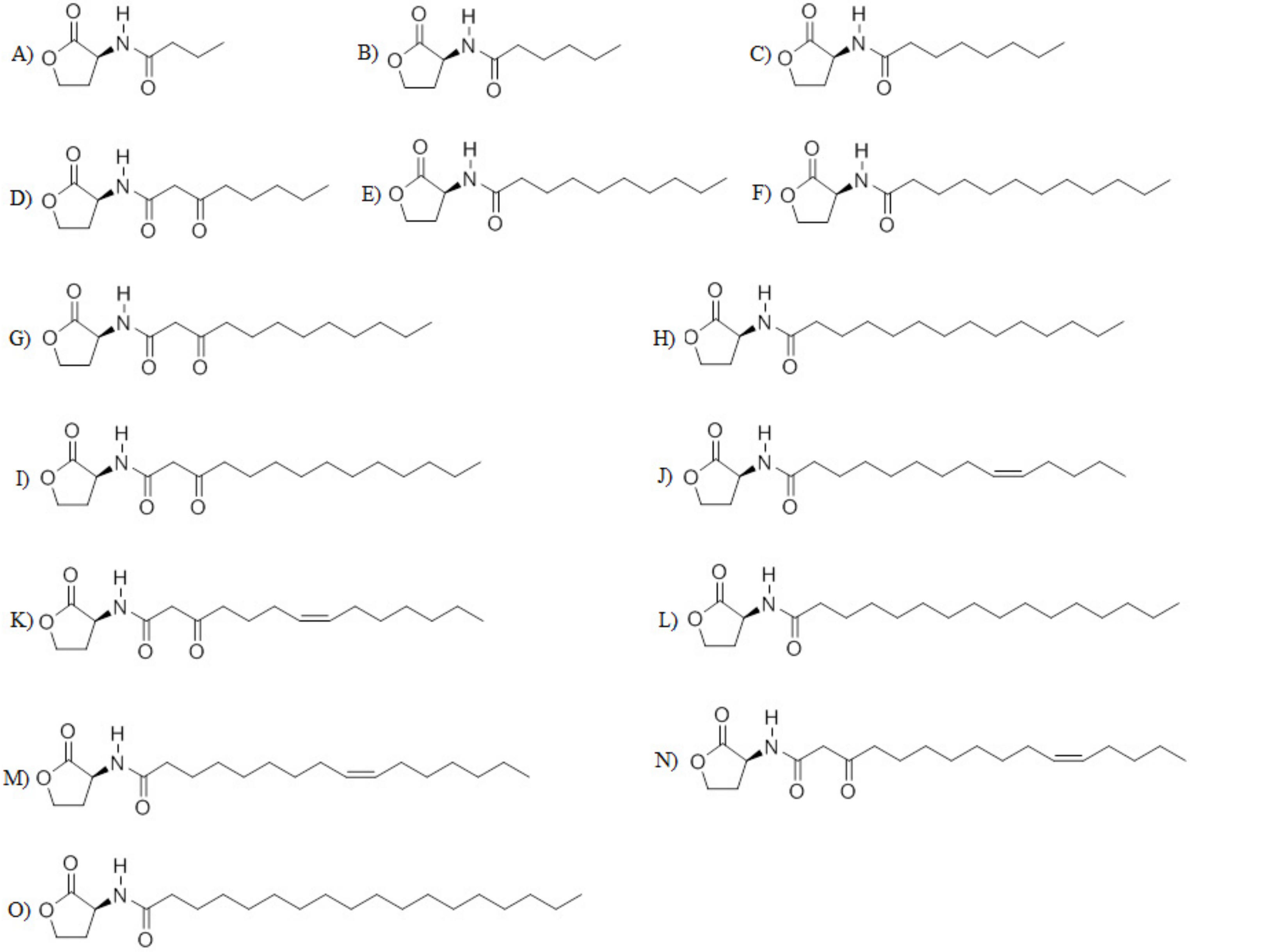

**Supplementary Figure 2**: Chemical structure of the AHLs used in this study **A)** $C_4$-HSL **B)** $C_6$-HL **C)** $C_8$-HSL **D)** 3-Oxo-$C_8$-HL **E)** $C_{10}$-HL **F)** $C_{12}$-HSL **G)** 3-Oxo-$C_{12}$-HSL **H)** $C_{14}$-HL **I)** 3-Oxo-$C_{14}$-HL **J)** C14:1-9-cis-(L)-HSL **K)** 3-oxo-C14:1-7cis-(L)-HSL **l)** $C_{16}$-HSL **M)** $C_{16:1}$-9 cis-(L)-HSL **N)** 3-Oxo-$C_{16:1}$-11 cis-(L)-HSL **O)** $C_{18}$-HSL.

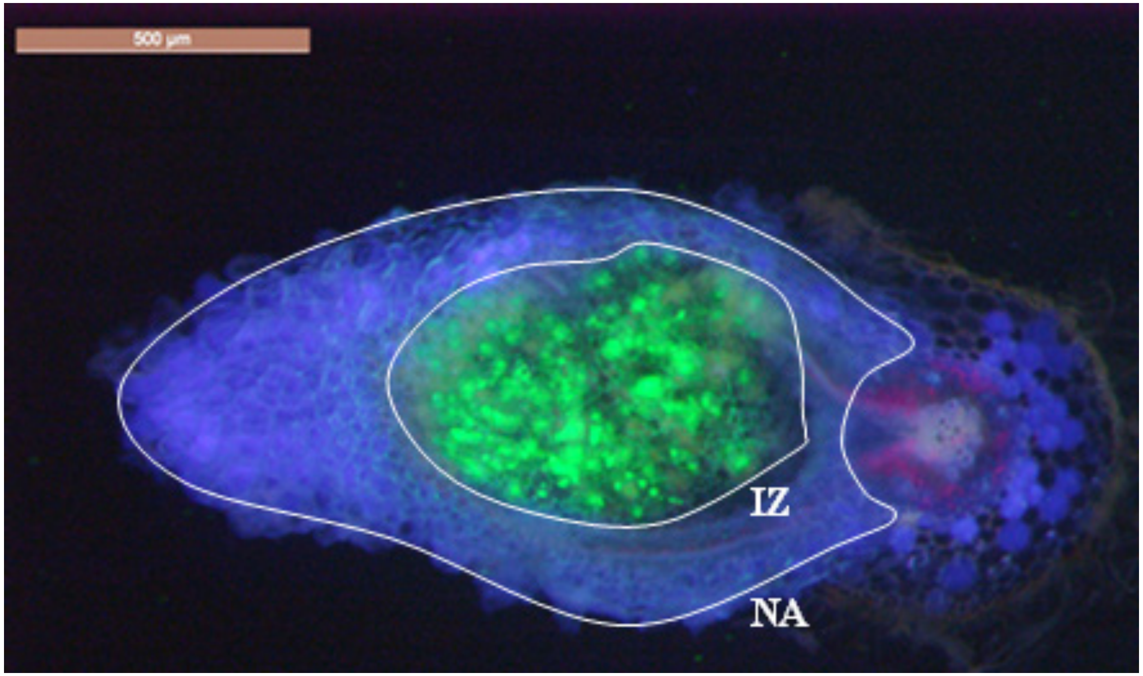


**Supplementary Figure 3**: Magnification of a nodule containing GFP labelled *S. meliloti* strain 1021 showing Nodule Area (NA) and Infection Zone (IZ) of 3 week-old *M. truncatula* seedlings.
